# Navigating Neurodegeneration: Integrating Biomarkers, Neuroinflammation, and Imaging in Parkinson’s, Alzheimer’s, and Motor Neuron Disorders

**DOI:** 10.3390/biomedicines13051045

**Published:** 2025-04-25

**Authors:** Masaru Tanaka, Simone Battaglia, Donato Liloia

**Affiliations:** 1 Danube Neuroscience Research Laboratory, HUN-REN-SZTE Neuroscience Research Group, Hungarian Research Network, University of Szeged (HUN-REN-SZTE), Tisza Lajos krt. 113, H-6725 Szeged, Hungary; 2 Center for Studies and Research in Cognitive Neuroscience, Department of Psychology “Renzo Canestrari”, Cesena Campus, Alma Mater Studiorum, University of Bologna, 47521 Bologna, Italy; simone.battaglia@unibo.it; 3 Department of Psychology, University of Turin, 10124 Turin, Italy; donato.liloia@unito.it

## 1. Introduction

Neurodegenerative diseases represent a daunting global challenge, affecting millions worldwide and imposing significant clinical and socioeconomic burdens [1,2,3]. Their multifaceted nature arises from intersecting genetic, metabolic, and environmental factors, culminating in progressive declines in cognition, motor control, and overall functionality [4,5,6]. Despite relentless scientific efforts, effective interventions remain elusive, underscoring the urgency for novel strategies and collaborative solutions. In this Topical Collection, titled “Neurodegeneration No More: Cutting-Edge Technologies and Therapies in the Evolution of Neurodegenerative Disease Management”, we present innovative research seeking to transform clinical outcomes (https://www.mdpi.com/journal/biomedicines/topical_collections/Neurodegeneration_No_More (accessed on 24 April 2025)). By examining emerging diagnostic tools, advanced therapeutic targets, and integrative care pathways, these articles address persistent gaps that hinder successful patient management [7,8,9,10,11]. Beyond mapping molecular mechanisms, these articles emphasize holistic models of care and patient-centered approaches with enhanced quality-of-life measures [12,13,14,15]. This collection of articles paves the way for a future where disease progression can be curbed or potentially reversed, offering renewed hope for individuals, caregivers, and the broader medical community [16,17,18]. Together, these articles highlight the necessity for sustained interdisciplinary cooperation and deeper investment in ongoing research [19,20,21].

Despite steady progress in neurodegenerative research, multiple critical gaps remain [22,23,24]. Identifying robust biomarkers to track disease progression is challenging, particularly when motor dysfunction intersects with neuroinflammatory processes [25,26,27]. The goal to clarify genotype–phenotype links and develop effective, individualized interventions remains a significant challenge in the context of movement and motor neuron disorders [28,29,30]. Meanwhile, reliable methods to detect subtle neuropathological changes early in Alzheimer’s disease (AD) and related dementias have yet to be developed, limiting opportunities for timely therapeutic action [31,32,33,34,35]. Similarly, neurovascular and neuroinflammatory conditions demand more research into how endothelial dysfunction, blood–brain barrier permeability, and local immune responses evolve and interact [36,37,38,39]. Across these domains, translating novel diagnostics and therapeutic breakthroughs from bench to bedside calls for larger, more diverse cohorts to validate findings [40,41,42,43]. Refined imaging modalities and big data analytics also hold promise but require further development for precise patient stratification [44,45,46,47]. Additionally, the interplay between comorbidities and mental health remains underexplored, underscoring the need for integrative approaches [45,48,49,50,51]. Finally, constrained funding poses significant barriers to the long-term research needed to develop new treatment paradigms [48,52,53].

This collection brings innovative discoveries together with real-world patient care under a unified framework, encouraging collaboration among researchers, clinicians, and industry partners [54,55,56]. By emphasizing advanced diagnostics, personalized therapeutic approaches, and the integration of diverse expertise, these articles deepen our understanding of disease mechanisms and help shape the future standard of care [57,58,59]. Each study addresses a particular issue—whether in Parkinson’s disease, other movement and motor neuron disorders, dementia, or neurovascular and inflammatory conditions—yet collectively underscores the necessity of interdisciplinary solutions [60,61,62]. Highlighting both recent breakthroughs and unresolved questions, the contributions stress the value of exploring molecular drivers, harnessing innovative technologies, and prioritizing patient-centric strategies for improved outcomes [63,64,65]. As new insights coalesce across multiple domains, this issue bridges clinical practice and cutting-edge research, prompting the development of meaningful interventions in the field [63,64,66]. Ultimately, the collaborative spirit reflected in this collection aims to accelerate the transformation of foundational knowledge into tangible impacts, fostering hope for individuals grappling with neurodegenerative disease [66,67,68].

## 2. Topical Collection Articles

This section presents 17 innovative papers advancing our understanding of complex neurological disorders, including Parkinson’s disease, motor neuron disorders, and dementia (Table 1). They examine metabolic and autonomic facets of Parkinson’s, explore gene-targeted and circadian therapies in movement disorders, and unveil promising non-invasive interventions for mild cognitive impairment. Novel biomarker discoveries, neuroimaging techniques, and anti-inflammatory approaches are also highlighted, reflecting the potential for personalized treatments. Collectively, these findings lay the foundation for more precise and effective management of neurodegenerative diseases.

### 2.1. Parkinson’s Disease

Parkinson’s disease continues to fascinate researchers, demanding new strategies to unravel its complexities [69,70]. The first paper delves into alpha-synuclein aggregation and mitochondrial dysfunction while offering a bold perspective that links disturbed lipid–glucose metabolism, insulin resistance, and glycolipid insults, suggesting that antidiabetic agents might slow disease progression [71]. Another study explores autonomic dysfunction, revealing that repeated whole-body cryostimulation enhances parasympathetic activity and heart rate variability while preserving blood pressure, revealing a novel route to rebalance the sympathovagal dynamics of Parkinson’s disease [72,73]. A multi-method approach then positions clozapine as the leading choice for Parkinson’s psychosis, with pimavanserin as an alternative, supported by systematic reviews and surveys that confirm clozapine’s effectiveness and tolerability [74]. Meanwhile, new insights into alpha-synuclein pathology reinforce its dual role as both a biomarker and a therapeutic target, fueling the development of interventions in the near future that can alter disease progression [75]. Finally, translational evidence shows that β2-adrenergic agents, such as levalbuterol, reduce alpha-synuclein formation and inflammatory mediators in a manner similar to glucocorticoids, presenting a promising opportunity for broader applications across neurodegenerative and oncologic contexts [76]. These innovative findings herald an era of renewed hope for deciphering and ultimately conquering Parkinson’s disease [77].

**Table 1:** Thematic categories covering the topical collection “Neurodegeneration No More: Cutting-Edge Technologies and Therapies in the Evolution of Neurodegenerative Disease Management”.

Thematic Category		References
Parkinson’s Disease		
	(a) Metabolic Dysfunction in Parkinson’s Disease	[71]
	(b) Autonomic Modulation in Parkinson’s Disease with Whole-Body Cryostimulation	[72]
	(c) Treatment of Parkinson’s Disease Psychosis	[74]
	(d) The Neurobiology and Potential of α-Synuclein in Parkinson’s	[75]
	(e) Beta2-Adrenergic Suppression of Neuroinflammation in Parkinsonism	[76]
2. Other Movement and Motor Neuron Disorders		
	(a) Nusinersen in Spinal Muscular Atrophy (Types 2 and 3)	[78]
	(b) Circadian Interventions in Huntington’s Disease	[79]
	(c) Preserving Vocal Function in Amyotrophic Lateral Sclerosis via PDT and Adjuvant Therapies	[80]
	(d) Mitochondrial Dysfunction in Sporadic Amyotrophic Lateral Sclerosis	[81]
3. Alzheimer’s Disease and Related Cognitive Disorders		
	(a) Transcranial Pulse Stimulation in Mild Neurocognitive Disorder	[82]
	(b) Tau PET/CT Imaging in Alzheimer’s Disease and Frontotemporal Lobar Degeneration	[83]
	(c) Antidepressants and Serotonin Receptors in Aβ-Oligomer Neurotoxicity	[84]
4. Neurovascular and Neuroinflammatory Conditions		
	(a) Cerebral Microbleeds, Blood–Brain Barrier Dysfunction, and Stroke Risk	[85]
	(b) EAE without Pertussis Toxin: Transcriptome Insights into Multiple Sclerosis	[86]
5. Novel Diagnostic, Therapeutic, and Biomarker Approaches		
	(a) Psychiatric Burden in Glaucoma Patients	[87]
	(b) rTMS and TBS for Neurotransmitter Modulation, Receptor Dynamics, and Neuroimaging	[88]
	(c) Oxytocin and Vasopressin Gene Expression as Potential Biomarkers for Cannabidiol Efficacy	[89]

EAE, Experimental Autoimmune Encephalomyelitis; PDT, percutaneous dilatational tracheostomy; PET/CT, positron emission tomography/computed tomography; rTMS: repetitive transcranial magnetic stimulation; TBS, theta-burst stimulation.

### 2.2. Other Movement and Motor Neuron Disorders

Neuromuscular disorders demand innovative solutions, and these four studies offer powerful new insights [90]. Over 54 months, nusinersen substantially boosted motor function in spinal muscular atrophy (SMA) types 2 and 3, with Generalized Estimating Equation analysis pinpointing time, SMA type, age, and exon deletions as key factors—underscoring the importance of personalized interventions [78]. Huntington’s disease research reveals that circadian imbalances contribute to worsening motor and cognitive decline, but restoring normal sleep/wake patterns could slow progression and unlock new clinical possibilities [79]. Meanwhile, combining percutaneous dilatational tracheostomy with targeted adjuvant therapies helped some patients with limb-type amyotrophic lateral sclerosis (ALS) retain vocal function, though results varied, calling for further investigation [80]. Finally, high-resolution respirometry in sporadic ALS showed reduced complex I and II function in peripheral blood mononuclear cells, highlighting mitochondrial dysfunction as a crucial driver and a promising diagnostic/therapeutic focus [81]. These groundbreaking findings fuel renewed hope for tackling these challenging conditions.

### 2.3. Alzheimer’s Disease and Related Cognitive Disorders

As these three studies demonstrate, neurogenerative disorders are primed for new solutions [91]. In adults with mild neurocognitive disorder, transcranial pulse stimulation boosted cognition and shifted functional connectivity, suggesting a promising non-invasive therapy that warrants testing in larger-scale trials [82,92]. For 24 patients suspected of AD or frontotemporal lobar degeneration, [11C]PBB3 PET/CT imaging revealed tau pathology closely linked to cognitive decline, outperforming traditional biomarkers and offering a more precise diagnostic method [83]. Finally, comparing fluoxetine, duloxetine, and mirtazapine showed significant antioxidant effects, especially duloxetine, which counteracted Aβ oligomer-induced toxicity through 5-HT1A receptor-mediated pathways, hinting at the potential of antidepressants to slow AD [84]. These findings energize ongoing efforts to combat and possibly reverse cognitive decline [93,94].

### 2.4. Neurovascular and Neuroinflammatory Conditions

Neurovascular integrity is a central challenge in neuroscientific research, as demonstrated by these two studies. Cerebral microbleeds, which stem from vascular and blood–brain barrier dysfunction, heighten the risk of stroke and dementia and demand further research into microbleed pathogenesis to lessen the burden on aging populations [85,95]. Meanwhile, by inducing experimental autoimmune encephalomyelitis without pertussis toxin, the second study uncovers a slower, milder condition that closely aligns with human multiple sclerosis (MS). Transcriptomic data confirm strong MS-like signatures—Th1/Th2/Th17 activation—highlighting the model’s enhanced physiological relevance [86]. These findings present new possibilities to better diagnose and manage vascular and inflammatory brain disorders.

### 2.5. Novel Diagnostic, Therapeutic, and Biomarker Approaches

Neurological disorders often demand a broad and integrated approach, as confirmed by these three papers. A systematic review of 29 studies highlights the high prevalence of depression and anxiety in glaucoma, calling for multidisciplinary collaboration to address both ocular health and mental well-being [87]. Another review reveals that repetitive transcranial magnetic stimulation (rTMS) and theta-burst stimulation (TBS) methods boost neuroplasticity via GABAergic, glutamatergic, and BDNF-TrkB pathways, suggesting that personalized stimulation protocols could enhance recovery in complex neurological diseases [88,96]. Meanwhile, new findings show that cannabidiol (CBD) modestly increases oxytocin and vasopressin gene expression, even under inflammatory conditions, pointing to their potential role as biomarkers and reinforcing CBD’s anti-inflammatory benefits [89]. These advances underscore the importance of innovative, holistic strategies for diagnosing and managing neurological challenges.

## 3. Discussion

This Topical Collection bridges fundamental gaps, highlighting new possibilities for diagnosis, treatment, and patient management. Research on Parkinson’s delves into the interplay between metabolic dysfunction, autonomic modulation, and psychosis, suggesting how specific interventions can target non-motor elements of the disease [71,72,74,75,76]. It also illustrates advancements in movement and motor neuron disorders, demonstrating the efficacy of nusinersen, circadian modulation, and improved tracheostomy techniques [78,79,80,81]. In dementia-focused studies, the integration of tau PET/CT imaging and transcranial pulse stimulation points to more precise diagnostic approaches and potential therapeutic breakthroughs [82,83,84]. Within neurovascular and neuroinflammatory contexts, these works underline associations between cerebral microbleeds, immune signaling, and disease progression, shedding light on nuanced therapeutic avenues [85,86]. Importantly, the exploration of rTMS, TBS, and biomarker-based strategies highlights the promise of personalized approaches that factor in psychiatric symptoms, receptor dynamics, and gene expression [87,88,89,97]. By compiling pilot data, systematic reviews, and cross-disciplinary insights, the authors emphasize the importance of holistic models of care that integrate next-generation methods and improve patient-centric outcomes. Their collective emphasis on translational applications promotes improvements in therapeutic efficacy.

From a theoretical standpoint, these studies expand upon established frameworks in neurodegeneration research by combining molecular biology, biomedical engineering, and clinical insights [98,99]. This multidisciplinary perspective enriches our comprehension of pathophysiological mechanisms while presenting new directions for therapy development [100,101]. Practically, their findings reaffirm the necessity of individualized treatment plans, particularly for chronic and progressive conditions. Nevertheless, some studies are constrained by limited sample sizes or observational designs, demonstrating the need for larger, long-term trials to validate these promising findings [102,103].

Overall, the clinically relevant nature of these investigations stresses real-world applicability. By prioritizing patient experiences and harnessing the synergy between academic research, technological advances, and clinical execution, this Topical Collection brings new momentum to an evolving field [50]. These efforts will hopefully inspire future endeavors, ultimately resulting in the development of meaningful interventions that alleviate the global burden of neurodegenerative diseases.

## 4. Conclusions

The contributions within this Topical Collection underscore the power of integrating diverse scientific methods and clinical insights to advance neurodegenerative disease management. By addressing key gaps in early detection, disease mechanisms, and therapeutic interventions, these studies highlight novel pathways toward precision medicine. Their cross-cutting themes—spanning biomarker discovery, neuromodulation, and metabolic optimization—reinforce the need for multifaceted approaches that transcend traditional disciplinary boundaries. Collectively, they foster a vision of patient-centered care that harnesses big data, cutting-edge imaging, and innovative pharmacological targets to mitigate or reverse disease progression. Moreover, by emphasizing non-motor symptoms and comorbidities, the findings remind us that effective management must account for the entire spectrum of patients’ experiences. As our knowledge expands, so does the potential for clinical breakthroughs that can significantly enhance quality of life. This Topical Collection thus offers a platform to guide future translational research and promote meaningful shifts in global healthcare practices [104].

## Abbreviations

The following abbreviations are used in this manuscript:

AD	Alzheimer’s disease
ALS	amyotrophic lateral sclerosis
CBD	cannabidiol
MS	multiple sclerosis
PDT	percutaneous dilatational tracheostomy
PET/CT	positron emission tomography/computed tomography
rTMS	repetitive transcranial magnetic stimulation
SMA	spinal muscular atrophy
TBS	theta-burst stimulation
